# Mangrove Health Index, Community Structure and Canopy Cover in Small Islands of Bunaken National Park, Indonesia: Insights into Dominant Mangrove Species and Overall Mangrove Condition

**DOI:** 10.21315/tlsr2024.35.2.9

**Published:** 2024-07-31

**Authors:** Joshian Nicolas William Schaduw, Trina Ekawati Tallei, Deiske A Sumilat

**Affiliations:** 1 Department of Marine Science, Faculty of Fisheries and Marine Science, Sam Ratulangi University, Manado 95111, Indonesia; 2 Department of Biology, Faculty of Mathematics and Natural Science, Sam Ratulangi University, Manado 95111, Indonesia

**Keywords:** Mangrove Ecosystem, Mangrove Health Index, Community Structure, Bunaken National Park

## Abstract

Mangrove ecosystems are crucial for protecting littoral regions, preserving biodiversity and sequestering carbon. The implementation of effective conservation and management strategies requires a comprehensive understanding of mangrove community structure, canopy coverage and overall health. This investigation focused on four small islands located within the Bunaken National Park in Indonesia: Bunaken, Manado Tua, Mantehage and Nain. Utilising the line transect quadrant method and hemispherical photography, the investigation comprised a total of 12 observation stations. Nain had the greatest average canopy coverage at 76.09%, followed by Mantehage, Manado Tua and Bunaken at 75.82%, 71.83% and 70.01%, respectively. Mantehage had the maximum species density, with 770.83 ind/ha, followed by Bunaken, Nain and Manado Tua with 675 ind/ha, 616.67 ind/ha and 483.34 ind/ha, respectively. The predominant sediment type observed was sandy mud and the mangrove species identified were *Avicennia officinalis* (AO), *Bruguiera gymnorrhiza* (BG), *Rhizophora apiculata* (RA), *R. mucronata* (RM), and *Sonneratia alba* (SA). On the small islands, *S. alba* emerged as the dominant mangrove species based on the importance value index (IVI). In addition, the Mangrove Health Index revealed that only 6.79% of the region exhibited poor health values, while 50% of the region was categorised as being in outstanding condition. These findings indicate that the overall condition of mangroves on these islands was relatively favourable.

**Highlights**

- The health condition of the mangrove community can be considered relatively good, falling within the moderate category as indicated by the Mangrove Health Index (MHI) values. Approximately 6.79% of the area displays poor health condition, whereas 50% of the area was classified as being in excellent condition.
- *Sonneratia alba* species demonstrated the highest Importance Value Index (IVI), while *Rhizophora apiculata* species exhibited the lowest IVI.
- The mangrove community on these islands encompasses five different species, namely *Avicennia officinalis, Bruguiera gymnorrhiza, R. apiculata, R. mucronate* and *S. alba*. The mangrove density ranged from 483.34 ind/ha to 770 ind/ha, with the average canopy cover falling between 70.04% and 76.09%.

## INTRODUCTION

The global extent of mangrove ecosystems is estimated at 15 million hectares (ha), providing habitat for diverse marine organisms and offering various benefits to human populations (Carugati *et al*. 2018). Indonesia harbours the largest mangrove ecosystem worldwide, covering 22.6% of the total global area. The Indonesian Ministry of Environment and Forestry reported that the country’s mangrove area spaned approximately 3.36 million ha (Rahadian *et al*. 2019). This extensive distribution can be attributed to Indonesia’s geographic location in the tropics, second-longest coastline globally and flat coastal geomorphology, which favour the growth of mangroves on land and small islands (Nugroho *et al*. 2019; Kusmana *et al*. 2020; Dharmawan & Pramudji 2020; Insani *et al*. 2020).

Within the mangrove ecosystem, litter plays a fundamental role as the primary component of the food chain. Litter comprises plant leaves, branches, fruits and stems, which are decomposed by microorganisms, resulting in detritus particles that serve as a food source for filter-feeding aquatic organisms. The productivity of mangrove litter was estimated to be 7 to 8 tonnes per year per hectare (Alongi *et al*. 2002; Holmer & Olsen 2002). Mangroves thrive in intertidal areas and exhibit adaptability to salinity. They also interact with both fresh and seawater, forming a cohesive ecosystem that supports the survival of associated biota, including aquatic and terrestrial flora and fauna (Lugo & Snedaker 1974; Friess 2016a; Romanach *et al*. 2018; Martin *et al*. 2019; Macintosh 1991).

The services provided by mangrove ecosystems are vital for human well-being; however, these services are increasingly threatened by the impacts of climate change (Friess 2016b). While mangrove forests are globally recognised as highly productive coastal ecosystems, they are also vulnerable to human disturbances (Elith & Leathwick 2009; Lee *et al*. 2019). Additionally, mangroves form complex topographic systems that provide habitats alongside seagrasses and coral reefs, offering natural protection against erosion and tidal flooding. However, these systems are becoming increasingly susceptible to anthropogenic effects and have suffered degradation in several locations (Beck *et al*. 2011; Duarte *et al*. 2013; Narayan *et al*. 2016; Goldberg *et al*. 2020). Other anthropogenic pressures include urban development, agricultural activities leading to fertiliser and pesticide use, eutrophication, overfishing and heavy metal pollutants. Furthermore, natural disasters and the threat of climate change pose significant risks to the habitat functions of mangrove ecosystems (Brent *et al*. 2015; Hashim & Hughes 2010; Barbier *et al*. 2008). Ecological services provided by the coastal ecosystems including the mangrove, seagrass and coral reef of Indonesia, support livelihoods of many (Husain *et al*. 2020). As climate change leads to rising sea levels, mangroves play a crucial role in protecting small islands, making them an essential ecosystem.

Despite Indonesia having the world’s largest mangrove ecosystem, it is not exempted from high threats, with a decrease in mangrove area of approximately 140,000 ha since 2012. Mangrove degradation in the country is one of the largest worldwide and has significant implications for climate change (Richards & Friess 2016; Ilman *et al*. 2016). The loss of mangrove ecosystems greatly impacts hydrodynamic and geomorphological conditions, affecting their growth (Hurst *et al*. 2015). Reduced water flow can lead to sediment accumulation, which is then stabilised by the mangrove root system (Duarte *et al*. 2013). Activities such as mangrove planting, restoration and protection are crucial for mitigating the effects of climate change and understanding the current conditions. Research on climate change events and their effects on natural ecosystems typically involves field and modeling studies (Alexander 2016; Grant *et al*. 2017; Shan *et al*. 2021).

Given the functions and challenges faced by the ecosystem in this particular location, a study focusing on mangrove health is necessary. The Mangrove Health Index (MHI) is a commonly used analysis to assess the overall health of mangrove ecosystems. It involves combining information from various health indicators, such as tree density, canopy cover, species diversity, and sedimentation rates. The MHI enables comparisons of mangrove health among different locations or regions. For example, it can be used to compare the health of mangrove forests in different countries or to identify areas where conservation and restoration efforts are most needed. Therefore, this study aims to analyse the MHI, community structure, and canopy cover of mangroves on the small islands of Bunaken National Park, including Mantehage, Bunaken, Nain and Manado Tua. Mantehage Island, Indonesia’s furthest island, is particularly important to study. The results will complement the database on the potential coastal resources of small islands. Consequently, comprehensive data on seagrass beds and coral reefs from the previous year can be combined to provide information on the condition of the mangrove ecosystem (Schaduw *et al*. 2020; Schaduw & Kondoy 2020). The findings from this study will inform policymakers in developing conservation and sustainable utilisation regulations for the mangrove ecosystem on the small islands of Bunaken National Park.

## MATERIALS AND METHODS

### Study Site and Determination of Sampling Unit

**Figure 1:**
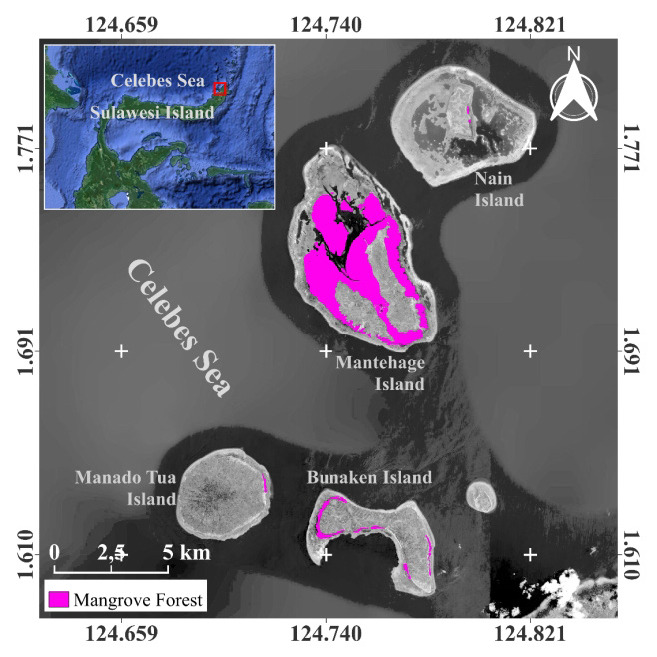
Map of mangrove distribution at study locations.

**Figure 2:**
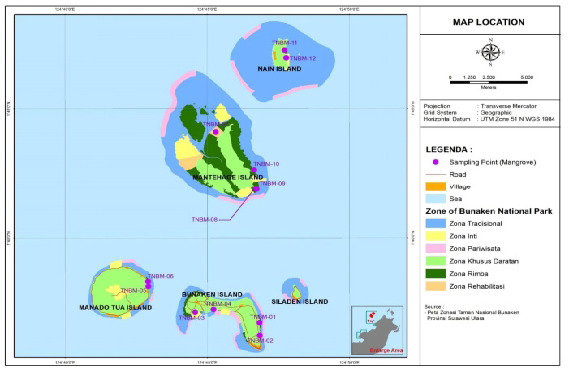
Study location and sampling point.

The present investigation was carried out within the small islands of Bunaken National Park, which encompass a mangrove ecosystems of the island Bunaken, Manado Tua, Mantehage and Nain. The study was conducted from August 2022 to May 2023. The research site was situated within two administrative regions of North Sulawesi Province, namely Manado City and North Minahasa Regency. Among the five small islands, only four were found to have a mangrove ecosystem, as depicted in Fig. 1. A total of 36 plots were established, distributed across 12 observation stations on each of the small islands, as illustrated in Fig. 2.

### Community Structure

Community structure data were obtained by conducting surveys within each 10 m × 10 m plot. The mangrove stem diameters were measured with the stratified purposive sampling method. A minimum of three plots were sampled within each zone, and the circumference of each mangrove stem was recorded for all trees with a diameter at breast height (DBH) of ≥ 16 cm. To mark each stem, spray paint with a width of less than 5 cm was used to encircle the tree. The measurements of stem circumference were then utilised to derive data on diameter (DBH), basal area, frequency, density, species dominance and the IVI. Additionally, satellite image analysis was employed to gather information on community structure and canopy cover, aiding in the determination of the area’s location and the overall community structure (Dharmawan, Suyarso, *et al*. 2020; Dharmawan, Hadi, *et al*. 2020). To identify all mangrove trees within each plot, reference books on mangrove identification were consulted (Tomlinson 1986; Kitamura *et al*. 1999; Noor *et al*. 1999; Giesen *et al*. 2006; Tomlinson 2016).

### Canopy Cover of Mangrove Communities

The hemispherical photography method was employed as one of the techniques to analyse canopy characteristics in the mangrove community. This method involves using photos taken through a wide-angle lens to estimate the amount of sunlight radiation and determine the percentage of plant cover (Anderson 1964). Hemispherical photos were captured using a smartphone camera with a resolution of 5 MP (Ptotal = 5,038,848 pixels), following the established requirements described by (Dharmawan, Suyarso, *et al*. 2020; Dharmawan, Hadi, *et al*. 2020). These photos were taken perpendicular to the sky, and each 10 × 10 m^2^ plot was divided into subplots or quadrants to determine the photo-taking positions based on the mangrove forest conditions. The percentage of mangrove canopy cover was calculated using the hemispherical photography method, which involved capturing photos at specific points (Jenning *et al*. 1999; Korhonen *et al*. 2006). Although relatively new for mangrove forests in Indonesia, this technique was easy to implement and provided more accurate data. The analysis involved separating the sky and vegetation pixels, and the percentage of vegetation canopy pixels was calculated using binary image analysis (Ishida 2004). In each plot, five photos were taken to obtain a representative sample, which was then analysed using ImageJ software to determine the number of pixels representing the canopy (P255). The percentage of canopy cover (C) in the mangrove community was calculated using Equation 1.


(1) $$\text{C}=\frac{\text{p}255}{\text{ptotal}}\times 100$$

where C = canopy cover; p255 = Konstanta canopy pixel and ptotal = pixel picture.

### Data Analysis

The collected data on canopy percentage, tree density, diameter and basal area were subjected to descriptive quantitative analysis to determine the mean values and standard errors for each zone. The mean values of these parameters and the IVI for each species across the entire mangrove area in the small islands of Bunaken National Park were calculated, taking into account the proportion in each zone (Dharmawan, Suyarso, *et al*. 2020). The Shapiro-Wilk normality test was conducted to assess the normal distribution of the data, followed by parametric analysis. Additionally, analysis of variance (ANOVA) followed by Tukey’s Honestly Significant Difference (HSD) test was performed on each parameter to identify differences in mean values among the zones.

For the interpolation of MHI values using remote sensing vegetation indices, linear regression analysis was conducted for each vegetation index. This analysis aimed to determine the best interpolation model for MHI values based on a single-band image. The Stepwise-Akaike Information Criterion (AIC) was used to evaluate the possible influence of multiple vegetation indices on the MHI value. The interpolation model with the highest regression coefficient (R^2^-adjusted) value was selected, and MHI distribution was mapped based on this model. The area of each MHI category, derived from the results of the best model interpolation, was calculated using QGIS software (Nurdiansah & Dharmawan 2021). The accuracy of the interpolation was assessed using the Root Mean Square Error (RMSE) method (Muhsoni *et al*. 2018).

### Mangrove Health Index (MHI) Analysis

The MHI serves as a valuable tool for monitoring changes in mangrove health over time and prioritising areas that require restoration and conservation efforts. Higher MHI values indicate a healthier mangrove ecosystem with improved ecological functioning, while lower values indicate ecosystem degradation or damage.

The MHI value for each plot was derived from three key components of the mangrove community structure parameters: the percentage scores of community canopy cover (SC), tree density (Snsp) and tree diameter (Sdbh). These components were calculated using Equations 2–5 as outlined in (Dharmawan, Suyarso, *et al*. 2020). To perform the MHI interpolation, a linear regression analysis was conducted to identify the most significant coefficient between MHI and remote sensing-based vegetation indices (Table 1). The satellite imagery used for this analysis was obtained from the Sentinel-2 satellite with the code L1C_T52MHE_A028485_20201205T013711 (Nurdiansah & Dharmawan 2021). Prior to the analysis, the satellite image underwent atmospheric and geometric correction using the Semi-Automatic Classification Plug-in (SCP) within the QGIS software, following the method described by Purwanto and Ardli (2020).


(2) Sc=0.25×c-13.06

(3) Snsp=0.13×Nsp+4.1

(4) Sdbh=0.45×DBH+1.42

(5) $$MHI=\frac{\left(Sc+Snsp+Sdbh\right)}{3}\times 10$$

where Sc = score value of community cover percentage; Snsp = sapling density; and SDBH = tree-spaling diameter.

## RESULTS

**Figure 3:**
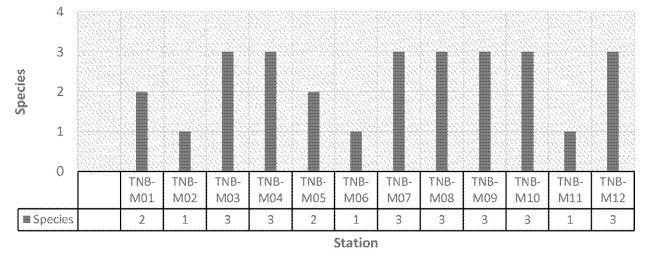
Number of mangrove types at each station.

The number of mangrove species varied among the islands, as illustrated in Fig. 3, Table 2 and Table 3. These species included *Avicennia officinalis* (AO), *Bruguiera gymnorrhiza* (BG), *Rhizophora apiculata* (RA), *Rhizophora mucronata* (RM) and *Sonneratia alba* (SA). Specifically, Bunaken and Mantehage had four mangrove species, Nain had three and Manado Tua Island had two. However, *S. alba* was present on all of the small islands, as depicted in Table 3.

**Figure 4:**
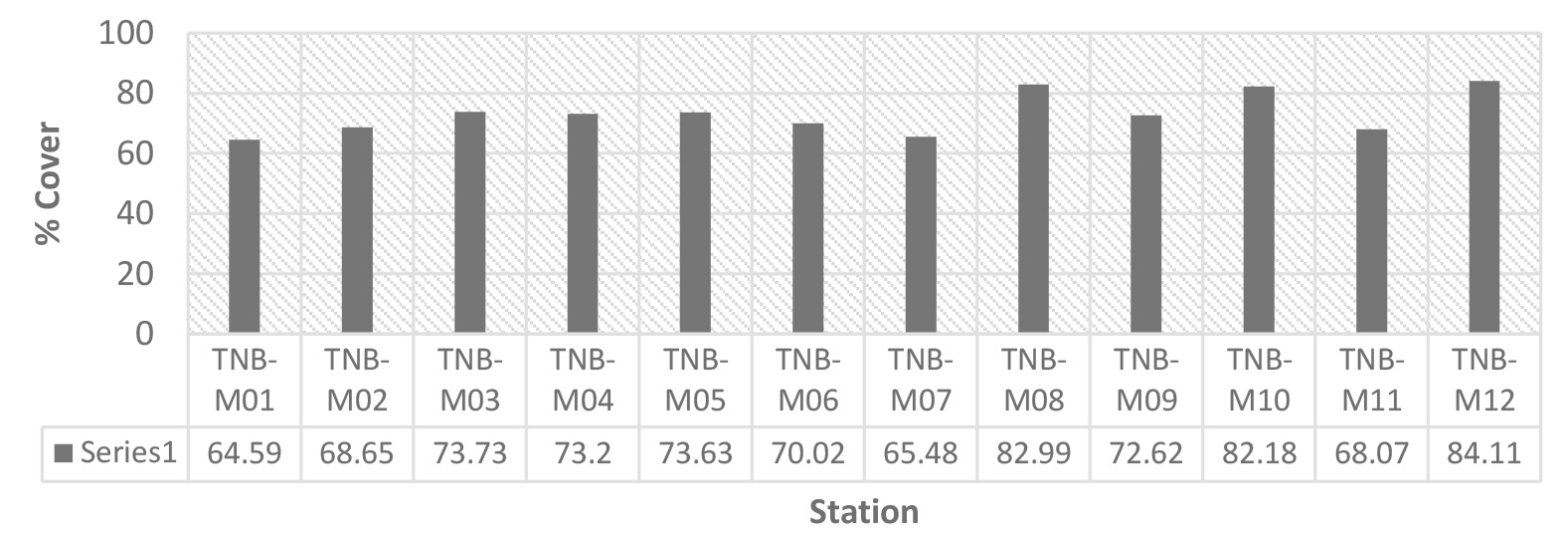
Percentage of mangrove tree density.

The percentage of canopy cover was assessed on various islands within Bunaken National Park. On Bunaken Island, the canopy cover ranged from 64.59% to 73.73%. Meanwhile, on Manado Tua Island, the range was between 70.02% and 73.63%. Mantehage Island and Nain Island exhibited canopy cover ranges of 65.48% to 82.99% and 68.07% to 84.11%, respectively, as presented in Table 4. Nain Island had the highest average percentage of canopy cover at 76.09%, followed by Mantehage, Manado Tua and Bunaken Island at 75.82%, 71.83% and 70.04%, respectively, as displayed in Fig. 4. The TNB-M12 station on Nain Island had the highest recorded canopy cover, while the TNB-M01 station on Bunaken Island had the lowest recorded canopy cover.

**Figure 5:**
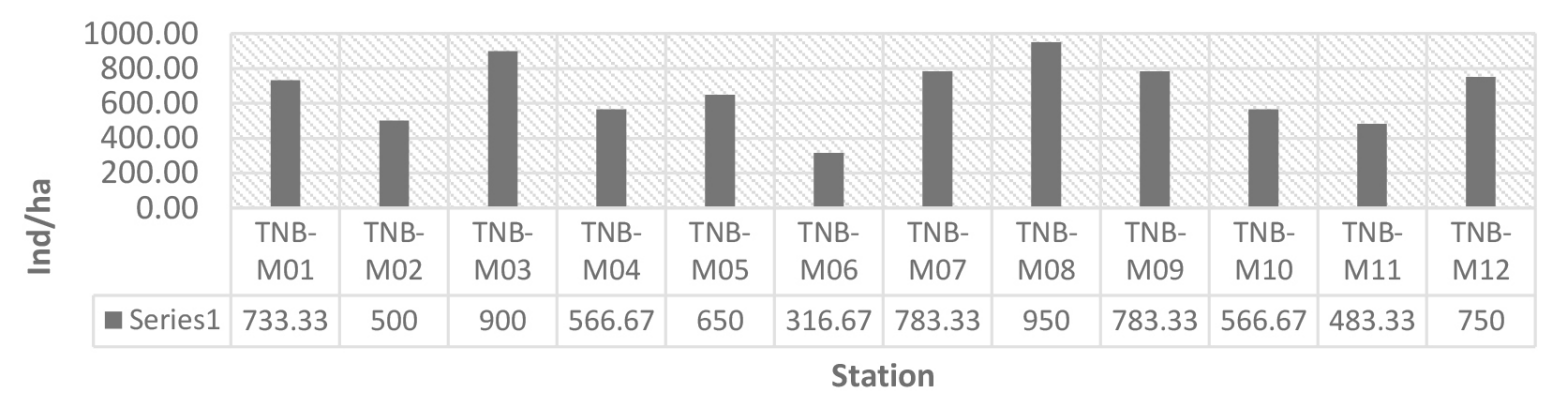
Mangrove importance value index.

The analysis results concerning the average density of mangrove trees reveal that Mantehage Island exhibited the highest value of 770.83 ind/ha. This was followed by Bunaken, Nain and Manado Tua, with densities of 675 ind/ha, 616.67 ind/ha and 483.34 ind/ha, respectively, as depicted in Table 4. Fig. 5 illustrates the density at each observation station, with TNB-M08 on Mantehage Island recorded the highest value of 950 ind/ha, while the lowest density of 316.67 ind/ha was observed at TNB-M06 on Manado Tua Island. In contrast, Anthoni *et al*. (2017) reported that the northern mainland of Bunaken National Park displayed the highest density in Tiwoho Village for *R. mucronata*, with a value of 1,330 ind/ha. On the other hand, the lowest density of 330 ind/ha was found in Bahowo Village for *B. gymnorrhiza* and *R. mucronata*. Mangrove density refers to the number of trees per unit area within a specific forest and varies based on factors such as mangrove species, environmental conditions and human activities.

The highest IVI for mangroves on Bunaken Island was observed for *S. alba*, while *R. mucronata* had the lowest IVI. On Manado Tua Island, *S. alba* had the highest IVI, while *R. apiculata* had the lowest. Mantehage Island possessed the largest mangrove ecosystem area, with the highest IVI attributed to *S. alba* and the lowest IVI associated with *R. apiculata*. In contrast, Nain Island displayed the highest IVI for *R. apiculata* and the lowest IVI for *S. alba*, as depicted in Table 4. Generally, *S. alba* exhibited the highest IVI among the small islands. In the northern mainland, *A. officinalis* had the highest IVI, while *R. mucronata* had the lowest IVI.

These variations in IVI values may be attributed to environmental factors specific to each study area, such as competition for nutrients, substrate conditions and variations in salinity levels, which can also influence the IVI and diversity index of mangrove species. The composition of the mangrove community is determined by several key factors, including substrate type, tidal conditions and salinity levels. In some cases, light availability and water movement also play important roles (Peng *et al*. 2016).

The obtained IVI values signify the ecological importance of each species within the ecosystem. In the context of mangroves, species with higher IVI are considered more ecologically and economically valuable. Furthermore, IVI can aid in management and conservation efforts by identifying species that are crucial for the health and productivity of the mangrove ecosystem. For instance, species with high IVI can be prioritised for protection and restoration measures, while those with low IVI may be managed differently.

## MANGROVE HEALTH INDEX (MHI)

**Figure 6:**
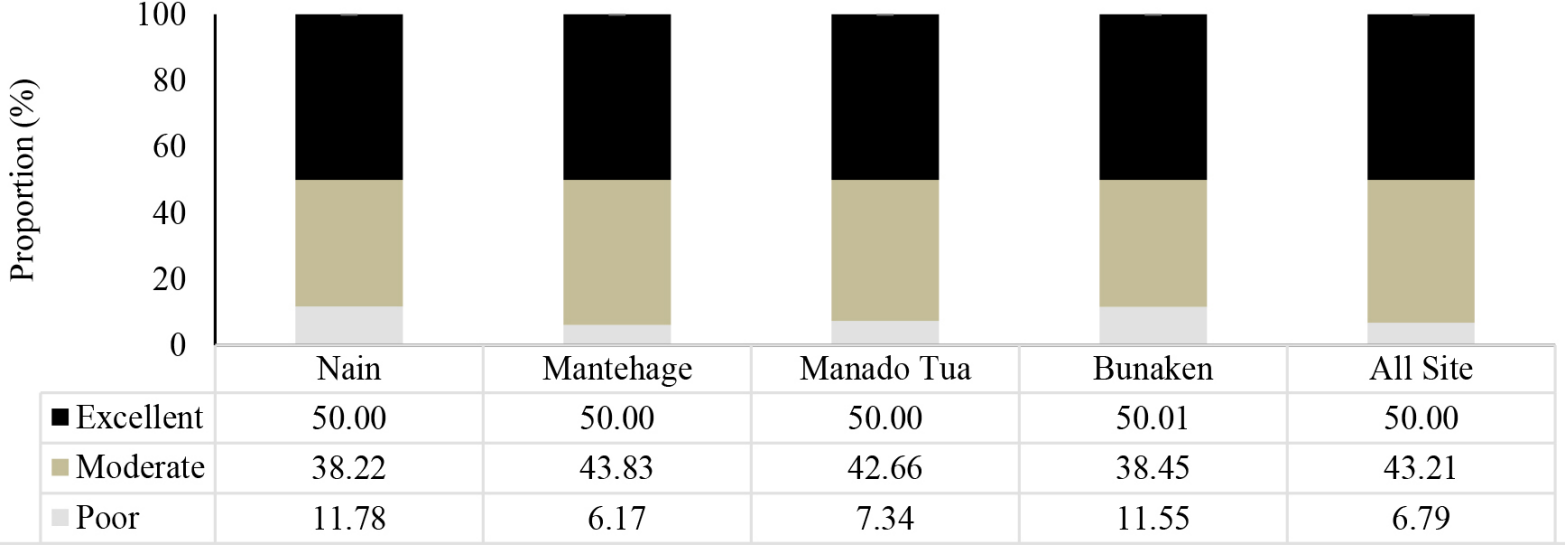
The proportion of mangrove health index for each island.

**Figure 7:**
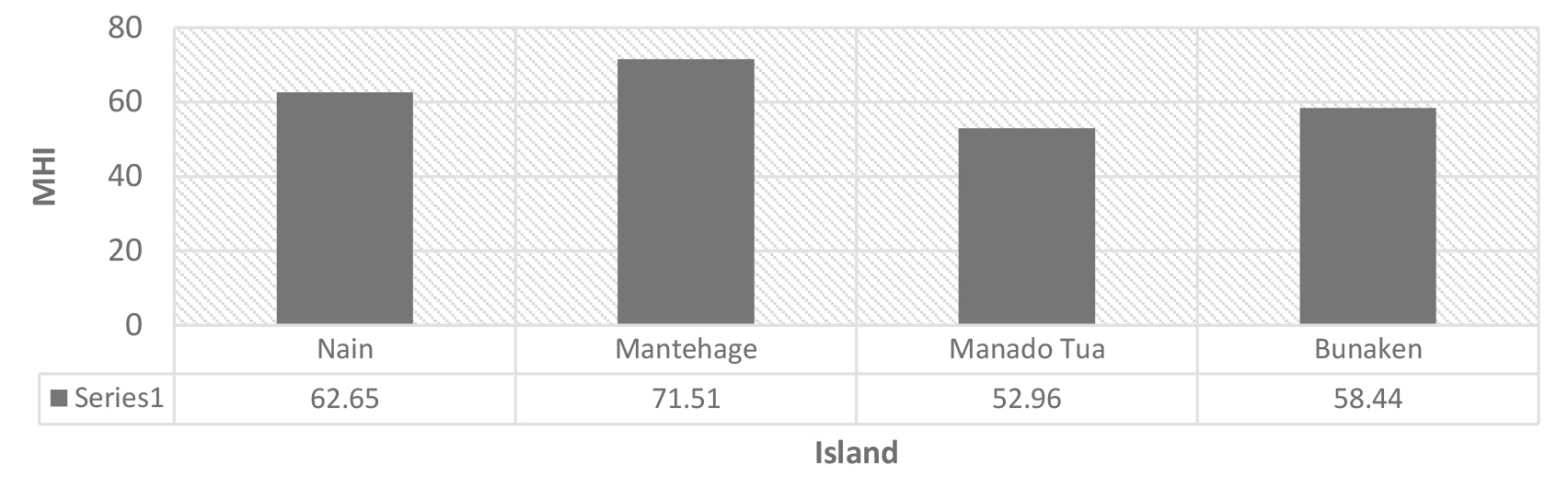
Mangrove health index for each island.

In general, the MHI in the small islands of Bunaken National Park can be classified as good, with an average proportional distribution of 50% excellent, 43% moderate and 6.69% poor, as illustrated in Fig. 6. The proportional range for excellent values was between 50% and 50.01%, for moderate values it was 38.22% to 43.83%, and for poor values, it ranged from 6.17% to 11.78%. The MHI varied among the different islands, with Mantehage Island having the highest value of 71.51, followed by Nain (62.65), Bunaken (58.44) and the lowest value was recorded in Manado Tua at 52.96, as shown in Fig. 7. These values indicate that the mangroves in the small islands were in good condition.

The results of linear regression analysis between remote sensing-based vegetation indices and MHI values indicate that the Mangrove Vegetation Index (MVI) exhibits the highest correlation with a regression coefficient of 0.71, compared to other individual indices, as presented in Table 5. MVI is a rapid and accurate method for identifying mangrove ecosystems using satellite imagery. This index incorporates information on greenness and moisture with 92% accuracy (Baloloy *et al*. 2020). However, a stronger relationship (R^2^-adjusted = 0.831) can be achieved by combining the values of the Normalised Burn Ratio (NBR), Green Chlorophyll Index (GCI), Structure Insensitive Pigment Index (SIPI) and Atmospherically Resistant Vegetation Index (ARVI). The regression coefficients for the first three vegetation indices were smaller than 0.50, as shown in Table 4. The interpolation values obtained are relatively accurate, as indicated by the root mean square error (RMSE) value of 4.46% or less than 5% (Table 5) (Nurdiansah & Dharmawan 2021). A lower RMSE value indicates that the employed formula is more effective in predicting actual values (Siddiq *et al*. 2020).

The mangrove ecosystem area in the small islands of Bunaken National Park encompassed a total of 748.53 ha. Among these, 374.27 ha were classified as being in excellent condition, 323.47 ha in moderate condition, and 50.79 ha in poor condition. Mantehage Island possessed the largest mangrove ecosystem area, covering 654.84 ha, with 327.42 ha in excellent condition, 286.99 ha in moderate condition, and 4.43 ha in poor condition. Bunaken Island followed with an area of 79.51 ha, comprising 39.76 ha in excellent condition, 30.57 ha in moderate condition, and 9.18 ha in poor condition. Manado Tua Island had an area of 11.04 ha, with 5.52 ha in excellent condition, 4.71 ha in moderate condition, and 0.81 ha in poor condition. Nain Island had the smallest area, measuring 3.14 ha, with 1.57 ha in excellent condition, 1.2 ha in moderate condition, and 0.37 ha in poor condition, as presented in Table 6.

## DISCUSSION

The diversity of mangroves in the small islands of Bunaken National Park is categorised as low due to the presence of only five species in this study. The level of diversity has a significant impact on the carbon absorption capacity of mangroves, with heterogeneous types demonstrating better carbon absorption compared to homogeneous types (Tinh *et al*. 2020). Dharmawan *et al*. (2020) observed that oceanic mangroves in small islands of Papua were predominantly dominated by *S. alba*, while Owi and Wundi Islands in Biak exhibited complete domination (IVI = 300%) due to the presence of a hard substrate type, consisting of sand and coral fragments. Despite their low canopy cover percentage, *S. alba* competes for space by producing allelopathic compounds that inhibit the growth of other mangrove species (Xin *et al*. 2013; Zhang *et al*. 2018). This pattern is similar to the mangroves in the northern part of Bunaken National Park, where six species were identified, namely *A. officinalis*, *Avicennia marina* (AM), *B. gymnorrhiza, R. apiculata, R. mucronata* and *S. alba* (Anthoni *et al*. 2017). Species distribution models can be utilised to assess the contributions of environmental variables and predict the spatial distribution of mangrove species (Austin *et al*. 2006; Merow *et al*. 2013; Rodriguez-Medina *et al*. 2020; Vessella & Schirone 2013).

Various factors influence the growth and distribution of mangroves, and these factors can be classified into several categories. These include sediment characteristics, physical and chemical attributes of water (such as temperature and salinity), climatic conditions (such as temperature and rainfall), tides, water quality, duration of flooding, coastline width and human activities related to land use (Giri *et al*. 2008; Forouzannia & Chamani 2022; Long *et al*. 2022). In addition to these factors, physiological characteristics of plants and other studies have identified 29 environmental variables that are utilised to predict suitable habitats for mangrove forests. These variables are grouped into four categories: bioclimate, terrain, water quality and hydrological conditions (Hu *et al*. 2020). These variables play a crucial role in determining the ecological conditions required for the establishment and persistence of mangrove ecosystems.

The average canopy cover of mangroves in the small islands of Bunaken National Park was recorded at 73.44%. This differs from the canopy cover observed in the mangroves of the northern mainland of Bunaken National Park. In the northern mainland, the highest canopy cover value was found in Meras Village, measuring 82.78%, while the lowest was recorded in Molas Village, with a value of 61.24%. Despite the variation, both areas can be categorised as having very dense canopy cover (≥ 75%) and being in good condition (Anthoni *et al*. 2017). Similarly, in comparable communities located on coral islands in Biak Regency, the percentage of canopy cover was approximately 61.32% (Dharmawan & Pramudji 2020). Another study conducted in Ayau Islands reported a relatively high percentage of mangrove canopy cover, ranging from 76.57% to 86.49% (Pribadi *et al*. 2020). Additionally, mangroves in Middleburg-Miossu Island, covering an area of 16.11 ha, exhibited relatively favourable community conditions. According to the classification outlined in Minister of Environment Regulation No. 201 of the Year 2004, the canopy cover percentage of mangrove communities in this island falls within the dense category (C ≥ 75%), with an average value of 75.82 ± 2.60% (Nurdiansah & Dharmawan 2021).

However, the extent of canopy cover has a significant impact on the condition of mangrove seedlings, as their survival ability diminishes considerably within a canopy cover range of 60%–90% (Jiang *et al*. 2019). Moreover, Nurdiansah and Dharmawan (2018) discovered a lower percentage of canopy cover (61.02%) in a mangrove community dominated by *S. alba* compared to communities dominated by Rhizophoraceae in the waters of Tidore and its surrounding areas, this matter pioneer species that thrives at the lower intertidal zone, with stronger wave and softer substratum. Conversely, the Rhizophoraceae mangrove community in the natural area of Wondama Regency exhibited a canopy cover percentage exceeding 75% (Dharmawan & Widyastuti 2017). The percentage of canopy cover directly influences light gaps and intensity, which is a factor that affects mangrove growth and regeneration (Peng *et al*. 2016). Additionally, tree size plays a vital role in assessing biomass and carbon dynamics, as well as ecosystem-level responses to environmental factors (Piponiot *et al*. 2022). Larger trees within the forest ecosystem significantly contribute to biomass and carbon stocks (Lindenmayer *et al*. 2012; Lutz *et al*. 2012; Ali *et al*. 2019).

The mean mangrove density in the small islands of Bunaken National Park was recorded as 636 individuals per hectare (ind/ha). Mangrove density serves as a crucial ecological indicator that reflects the overall health and productivity of the ecosystem. In general, mangrove forests with higher density are considered to be in a healthier and more productive state compared to those with lower density. This factor can also have implications for other important ecosystem functions and services, including carbon storage, coastal protection and habitat provision. Mangroves with higher density have the capacity to store more carbon per unit area, offer more effective protection against coastal erosion and storm events, and provide better habitats for a diverse range of marine species (Lindenmayer *et al*. 2012; Lutz *et al*. 2012; Ali *et al*. 2019; Piponiot *et al*. 2022; Tinh *et al*. 2020).

In certain instances, exceedingly high mangrove forest density can lead to overcrowding, resulting in resource competition for essentials such as light and nutrients. Consequently, this competition negatively impacts the growth and productivity of individual trees and ultimately leads to a decline in the overall health of the forest (Peng *et al*. 2016; Jiang *et al*. 2019).

**Figure 8:**
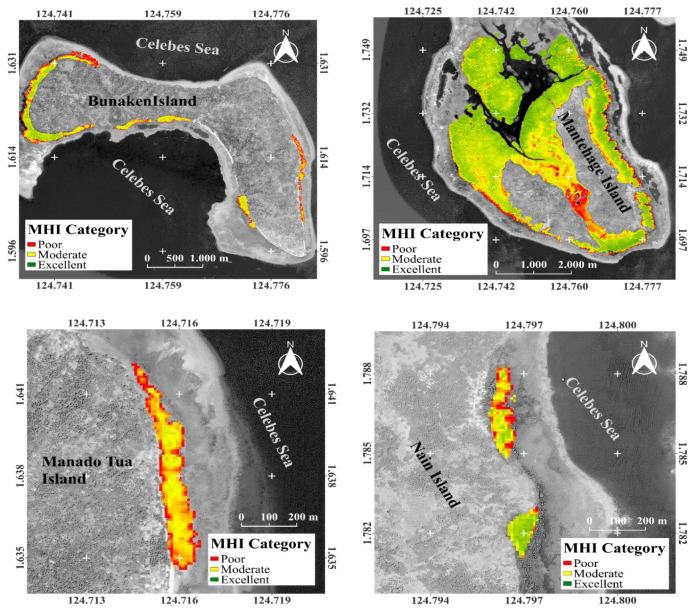
Interpolated mangrove health index distribution map.

Fig. 8 depicts the conditions of MHI for each island along with their proportional areas. These findings are consistent with the MHI observed in Molas Village, where the range fell between 48.66% and 69.79%, categorising it as “good.” Similarly, in Biak Numfor Regency, the MHI value was 65%, with a range of 39.3% to 76.8% (Dharmawan, Hadi, *et al*. 2020; Schaduw *et al*. 2021). On Middleburg-Miossu Island, less than 5% of mangroves exhibited poor health within the community. Utilising the interpolation method with the established formula, it was determined that the majority (55.73%) of mangroves were in the “moderate” health category, followed by 40.74% (6.56 ha) classified as “very good,” while only 3.53% were deemed to be in “poor” health (Nurdiansah & Dharmawan 2021). The ability of mangroves to attenuate wave energy is primarily influenced by the extent of forest area and the structural composition of the community (Bao 2011; Horstman *et al*. 2014).

The integration of remote sensing techniques with analysis of mangrove community structure and MHI has facilitated a more comprehensive assessment of mangrove health. The NBR index was employed to analyse the extent of mangrove areas, while the GCI index (Green Chlorophyll Index) was commonly used to estimate the chlorophyll content in leaves of various species, serving as an indicator of physiological and health conditions of the vegetation (Wu *et al*. 2012). The SIPI (Structure Insensitive Pigment Index) takes into account the ratio of carotenoids to chlorophyll, providing insights into mangrove health (Chaube *et al*. 2019). Additionally, the ARVI (Atmospherically Resistant Vegetation Index) exhibited a relatively high regression coefficient with MHI, and its correlation with mangrove carbon reserves in Teluk Benoa Bali was considered reasonably strong (Siddiq *et al*. 2020).

Tree density, diversity, evenness index and species richness are commonly used indicators for assessing mangrove health. However, these indicators may not provide stable measurements in homogeneous mangrove ecosystems, such as those found on small islands. In recent studies, satellite imagery has been utilised to evaluate the spatial quality of mangroves (Prasetya *et al*. 2017; Razali *et al*. 2019; Chougule & Sapkale 2020). The Mangrove Quality Index (MQI) has been developed to assess the overall quality of mangrove ecosystems based on the interrelationships between biotic, abiotic and socio-economic parameters. Nevertheless, the inclusion of complex parameters in the MQI presents challenges and requires significant resources (Faridah-Hanum *et al*. 2019). The MHI serves as a valuable tool for the conservation and management of mangrove ecosystems, providing a comprehensive assessment of their health status. The MHI can guide decision-making processes by informing the implementation of effective strategies for the protection and restoration of mangrove ecosystems and their associated ecological values.

The mangrove health index analysis method has certain limitations that should be acknowledged. Primarily, this method focuses primarily on the physical and structural characteristics of mangrove ecosystems, including tree density, canopy cover and stem diameter. It does not encompass other critical dimensions of mangrove health, such as biodiversity, ecosystem services and ecological processes. Consequently, a comprehensive evaluation of mangrove health necessitates the integration of additional indicators and metrics.

Another limitation pertains to the absence of a standardised protocol for conducting mangrove health index analysis. This lack of standardisation can result in inconsistent and unreliable outcomes across different studies. The absence of uniform guidelines makes it challenging to compare and contrast the health status of diverse mangrove ecosystems. Consequently, efforts to establish a standardised framework for conducting mangrove health index analyses are warranted to enhance the reliability and comparability of findings in future research endeavours.

## CONCLUSION

The small islands within Bunaken National Park, namely Mantehage, Bunaken, Nain and Manado Tua, possess distinct mangrove ecosystems. Among these islands, Mantehage Island harbors the largest mangrove ecosystem, whereas Manado Tua Island exhibits the smallest extent. The mangrove community on these islands encompasses five different species, namely *A. officinalis, B. gymnorrhiza, R. apiculata, R. mucronata* and *S. alba*. The mangrove density ranged from 483.34 ind/ha to 770 ind/ha, with the average canopy cover falling between 70.04% and 76.09%. Notably, *S. alba* species demonstrated the highest IVI, while *R. apiculata* species exhibited the lowest IVI. Overall, the health condition of the mangrove community can be considered relatively good, falling within the moderate category as indicated by the MHI values. Approximately 6.79% of the area displays poor health condition, whereas 50% of the area was classified as being in excellent condition. These findings collectively suggest that the mangrove condition on these islands was generally favourable.

## Figures and Tables

**Table 1:** Vegetation indices based on remote sensing analysis (Nurdiansah & Dharmawan 2021).

Vegetation indices ^Reference^	Formula
NDVI (Normalised Difference Vegetation Index)^1^	$\frac{\text{NIR}-\text{Red}}{\text{NIR}+\text{Red}}$
MI (Mangrove Index)^1^	$\frac{\text{NIR}-\text{SWIR}}{\text{NIR}\times \text{SWIR}}$
MVI (Mangrove Vegetation Index)^2^	$\frac{\text{NIR}-\text{Green}}{\text{SWIR}\times \text{Green}}$
SAVI (Soil Adjusted Vegetation Index)^1^	$\frac{\text{NIR}-\text{Red}}{\text{NIR}+\text{Red}+\text{L}}\times (1+\text{L})$
NBR (Normalised Burn Ratio)^1^	$\frac{NIR-SWIR}{NIR+SWIR}$
GCI (Green Chlorophyll Index)^1^	$\frac{\text{NIR}}{\text{Green}}-1$
EVI (Enhanced Vegetation Index)^1^	$\text{G}\times \frac{\text{NIR}-\text{Red}}{\text{NIR}+(\text{C}1\times \text{R})-(\text{C}2\times \text{Blue})+\text{L}}$
SIPI (Structure Insensitive Pigment Index)^1^	$\frac{\text{NIR}-\text{Blue}}{\text{NIR}-\text{Red}}$
ARVI (Atmospherically Resistant Vegetation Index)^1^	$\frac{\text{NIR}-2\times \text{Red}+\text{Blue}}{\text{NIR}+2\times \text{Red}+\text{Blue}}$

*Notes*: NIR = Near infrared; SWIR = Short-wave infrared; L = 1; G = 2.5, C1 = 6; C2 = 7.5.

(References: ^1^(Dharmawan, Suyarso, *et al*. 2020; ^2^Baloloy et al. 2020)

**Table 2:** Station, geographical coordinates, sediment and species.

No	Island	Local name	Station	Coordinate		Sediment	Number of species

				Long	Lat		
1	Bunaken	Bunaken Timur	TNB-M01	124°46′50,05″	01°36′45,31″	Muddy sand	2
		Bunaken Negeri	TNB-M02	124°44′34,28″	01°37′09,45″	Muddy sand	1
		Alung Banua	TNB-M03	124°46′50,34″	01°36′15,93″	Muddy sand	3
		Alung Banua	TNB-M04	124°45′12,89″	01°37′16,23″	Muddy sand	3
2	Manado Tua	Papindang	TNB-M05	124°42′55,40″	01°38′08,93″	Muddy sand	2
		Papindang	TNB-M06	124°42′54,83″	01°38′20,31″	Muddy sand	1
3	Mantehage	Buhias	TNB-M07	124°45′18,21″	01°44′06,23″	Muddy sand	3
		Tangkasi	TNB-M08	124°46′44,46″	01°41′53,26″	Muddy	3
		Tinongko	TNB-M09	124°46′42,17″	01°41′54,17″	Muddy sand	3
		Tinongko	TNB-M10	124°46′39,29″	01°42′38,98″	Muddy sand	3
4	Nain	Tarente	TNB-M11	124°47′43,11″	01°47′13,81″	Muddy sand	1
		Tarente	TNB-M12	124°47′46,85″	01°46′56,35″	Muddy sand	3

**Table 3:** Mangrove types on each island.

Species	Island			

	Bunaken	Manado Tua	Mantehage	Nain
*Avicennia officinalis* (AO)	×			
*Bruguiera gymnorrhiza* (BG)			×	
*Rhizophora apiculata* (RA)	×	×	×	×
*Rhizophora mucronata* (RM)	×		×	×
*Sonneratia alba* (SA)	×	×	×	×

**Table 4:** Canopy closure, density, and importance value index (IVI).

No	Island	Local name	% cover	Density (ind/ha)	IVI		% cover (average)	Density (average)

					Min	Max		
1	Bunaken	Bunaken Timur	64.59 ± 8.12	733.33 ± 70.33	RM 16.66	SA 283.34	70.04	675.00
		Bunaken Negeri	68.65 ± 5.29	500 ± 49.35		SA 300		
		Alung Banua	73.73 ± 15.08	900 ± 89.76	AO 18.42	SA 199.12		
		Alung Banua	73.20 ± 6.99	566.67 ± 163.30	RA 26.49	SA 218.54		
2	Manado Tua	Papindang	73,63 ± 3.91	650 ± 18,95	RA 16.59	SA 283.41	71,83	483,34
		Papindang	70.02 ± 9.61	316.67 ± 67.19		SA 300		
3	Mantehage	Buhias	65.48 ± 13.36	783.33 ± 39.18	RA 35.20	SA 140.67	75.82	770.83
		Tangkasi	82.99 ± 9.52	950 ± 116.37	RA 15.57	RM 177.40		
		Tinongko	72.62 ± 9.49	783.33 ± 215.06	RA 46.34	SA 130.24		
		Tinongko	82.18 ± 11.09	566.67 ± 36.53	BG 21.83	SA 163.16		
4	Nain	Tarente	68.07 ± 9.59	483.33 ± 100.60		RM 300	76.09	616.67
		Tarente	84.11 ± 8.11	750 ± 95.76	RM 14.51	RA 205.57		

**Table 5:** Linear models for predicting MHI value based on remote sensing vegetation indices, regression coefficient Adjusted R^2^ significance (F) and Accuracy-Test Value (RMSE).

Vegetation indices (X)	Formula: MHI (Y) =	R^2^-adjusted	F	RMSE
NDVI	84.81^*^NDVI + 16.709	0.631	30.068^***^	7.31
MI	−3.488^*^MI + 97.967	0.480	16.705^**^	10.25
MVI	28.367^*^MVI + 75.135	0.711	42.897^***^	6.46
SAVI	103.912^*^SAVI + 31.845	0.563	22.902^***^	7.95
NBR	209.780^*^NBR-79.158	0.481	16.749^**^	12.49
GCI	2.677^*^GCI + 45.22	0.384	11.577^**^	9.44
EVI	7.85^*^EVI + 41.965	0.389	11.803^**^	9.41
SIPI	−243.007^*^SIPI + 322.104	0.389	11.825^**^	9.40
ARVI	65.831^*^ARVI + 25.264	0.665	34.810^***^	6.96
NBR, GCI, SIPI, ARVI	102.12^*^NBR – 4.64^*^GCI + 178.15^*^SIPI + 159.53^*^ARVI - 252.39	0.831	21.8987^***^	4.46

*Notes*: NDVI = Normalised Difference Vegetation Index; MI = Mangrove Index; MVI = Mangrove Vegetation Index; SAVI = Soil Adjusted Vegetation Index; NBR = Normalised Burn Ratio; GCI = Green Chlorophyll Index; EVI = Enhanced Vegetation Index; SIPI = Structure Insensitive Pigment Index; ARVI = Atmospherically Resistant Vegetation Index

**Table 6:** Mangrove area and MHI condition in each island.

Area (ha)					

MHI category	Nain	Mantehage	Manado Tua	Bunaken	All site
Poor	0.37	40.43	0.81	9.18	50.79
Moderate	1.2	286.99	4.71	30.57	323.47
Excellent	1.57	327.42	5.52	39.76	374.27

Total mangrove (ha)	3.14	654.84	11.04	79.51	748.53

## Data Availability

Data will be made available on request.
